# Decompressive Surgery in Chemotherapy- and Radiotherapy- Induced Peripheral Compression Neuropathy: A Systematic Review

**DOI:** 10.3390/jcm15176538

**Published:** 2026-08-24

**Authors:** Tom Terng, Bas Schuitema, Mienke Rijsdijk, J. Henk Coert, Enrico Martin

**Affiliations:** 1 Department of Plastic and Reconstructive Surgery, University Medical Centre Utrecht, 3584 CX Utrecht, The Netherlands; 2 Department of Anaesthesiology, University Medical Centre Utrecht, 3584 CX Utrecht, The Netherlands; m.rijsdijk-2@umcutrecht.nl

**Keywords:** chemotherapy-induced peripheral neuropathy, radiation-induced peripheral neuropathy, treatment-related neuropathy, peripheral nerve compression, nerve entrapment, microsurgical decompression, peripheral nerve surgery

## Abstract

**Background**: Treatment-related peripheral neuropathies are disabling long-term complications of modern cancer therapy. Chemotherapy-induced peripheral neuropathy (CIPN) and radiation-induced peripheral neuropathy (RIPN) can lead to pain, motor deficits, and sensory loss, substantially impairing quality of life in cancer survivors. Surgical decompression has been proposed as a potential therapeutic option in cases of superimposed focal nerve compression due to treatment-related tissue changes, but evidence remains limited. This systematic review aims to evaluate the indications, surgical techniques, and outcomes of microsurgical decompression for cancer treatment-related peripheral neuropathies. **Methods**: A systematic search was performed in PubMed and Embase up to July 2026. Studies were screened by two independent researchers, and included when reporting outcomes of surgical intervention for chemotherapy-induced or radiation-induced peripheral neuropathy. Data on patient demographics, oncologic diagnosis, surgical indication, technique, and postoperative subjective and functional outcomes were extracted and presented in tables. **Results**: Fourteen studies met inclusion criteria describing 95 patients and 120 treated nerves. In CIPN, all surgically treated patients experienced pain relief and improvement in two-point discrimination, without reported deterioration. In RIPN, 88.5% of patients improved in pain, 58.8% in sensory function, and 44.4% in motor strength. Neurolysis combined with vascularized flap reconstruction was associated with better outcomes when compared with neurolysis alone. No major surgical complications were reported. Overall evidence quality was low, with small sample sizes and high heterogeneity among methodologies. **Conclusions**: Microsurgical decompression with or without vascularized flap reconstruction may provide meaningful pain relief and partial functional recovery in selected patients with treatment-related peripheral compression neuropathies refractory to conservative management. Nevertheless, current evidence is restricted to small retrospective series and case reports.

## 1. Introduction

Cancer remains one of the leading causes of mortality worldwide, with an estimated 20 million new cases each year worldwide [1,2]. Advances in screening, diagnosis, and treatment have significantly improved survival in many primary and metastatic malignancies [3]. However, these therapeutic successes have unveiled a growing clinical challenge in the burden of treatment-related toxicity. Among long-term survivors, neurological complications represent highly disabling sequelae of modern cancer therapy. Motor deficits and chronic neuropathic pain may persist for years, substantially impairing quality of life [4]. Iatrogenic induced neuropathies contain a heterogeneous group of symptoms differing in onset, progression, and prognosis. While early-onset complications are often transient, late-onset neuropathies tend to be progressive and are associated with irreversible functional loss [5]. As cancer survivorship continues to rise, recognition and management of treatment-induced neuropathy have become essential priorities in oncological care.

Chemotherapy-induced peripheral nerve compression is an underrecognized manifestation of systemic cancer treatment. Chemotherapy-related nerve injury may increase susceptibility to secondary compression neuropathies at anatomical sites of entrapment, potentially through treatment-induced tissue changes in the surrounding tissues. Clinically, patients present with distal neuropathic pain, paresthesia, numbness, and allodynia, often progressing to motor weakness and autonomic dysfunction at later stages [6,7]. Despite the high prevalence of chemotherapy-induced peripheral neuropathy (CIPN), affecting up to 68% of patients within one month after therapy and 30% beyond six months [8], the incidence of compression neuropathy specifically remains undefined. Currently, no effective treatment strategies exist, and management remains largely preventive or symptomatic [9]. Nevertheless, in specific cases, the underlying secondary fibrotic and compressive pathology suggests that surgical decompression may provide therapeutic benefit in selected patients.

Radiation-induced peripheral neuropathy (RIPN) represents a late complication of radiotherapy that is increasingly encountered in long-term cancer survivors. RIPN results from a combination of axonal injury and radiation-induced vascular damage. Radiation-induced brachial plexopathy (RIBP) is the most extensively described subtype and typically develops following irradiation of the chest wall, axilla, or neck. The reported incidence of RIBP is approximately 1.8–2.9% among patients receiving radiotherapy [10,11]. Clinically, RIBP presents with sensory disturbances, neuropathic pain, and progressive motor deficits. Current management strategies for RIBP remain largely symptomatic and focus on pain control [12]. However, in patients with persistent or progressive symptoms refractory to conservative therapy, surgical intervention may offer a therapeutic option rather than mere symptomatic relief. Several surgical techniques such as neurolysis, vascularized soft-tissue flaps, nerve grafting, and free functional muscle transfers have been reported to alleviate neuropathic pain, and in select cases, restore partial motor function [13,14]. Despite encouraging reports, current evidence is limited to small, heterogeneous case series, and surgical indications and outcome measures remain insufficiently defined. A clearer understanding of the feasibility, indications, and expected outcomes of surgical treatment is essential to guide clinical decision-making and improve long-term quality of life in this growing population of cancer survivors.

The objective of this review is to synthesize current evidence on surgical decompression, nerve reconstruction, and free function muscle transfer for chemotherapy- and radiation-induced compression neuropathies. We aim to assess their feasibility, indications, and outcomes in light of existing treatment paradigms.

## 2. Materials and Methods

This systematic review was conducted in accordance with the Preferred Reporting Items for Systematic reviews and Meta-Analyses (PRISMA) guidelines [15] and was not prospectively registered in any public registry. The completed PRISMA checklist is provided in the Supplementary Files S1 and S2.

### 2.1. Search Strategy

A systematic literature search was performed in PubMed and Embase databases up to February 2025 and was updated in July 2026. A search string was built using search terms related to “chemotherapy”, “radiotherapy”, “neuropathy”, and “surgical decompression.” The complete search syntaxes for PubMed and Embase are presented in Supplementary File S3. Additional relevant studies not identified through the syntax were identified from the reference lists of the included articles.

### 2.2. Eligibility Criteria

Studies were included if they met the following criteria: (1) patients with clinical symptoms of chemo- and/or radiotherapy-induced neuropathy of all ages; (2) patients treated by any form of surgical decompression; (3) evaluation of objective or subjective outcomes after surgery; (4) Articles published in the English or Dutch language. Exclusion criteria included: no full-text access, languages other than English or Dutch, animal studies, meta-analyses, (systematic) reviews, narrative reviews, expert opinions, and non-academic publications.

### 2.3. Selection Process and Data Synthesis

The initial screening and assessment of the articles based on title and abstract were done by two independent researchers (T.T and B.S) using Rayyan (Rayyan Systems Inc, Cambridge, MA, USA). Disagreements were solved through discussion. A full-text review of the potentially eligible studies was then performed by both. Data were extracted at an individual patient level, when available, and included sex, age, nerve treated in upper or lower extremity, tumor location, treatment modality (radiotherapy and/or chemotherapy), radiotherapy dose, chemotherapeutic agent, onset of symptoms, interval between therapy and intervention, complications, surgical intervention, functional outcomes, and length of follow-up.

Results were summarized and stratified according to the intervened peripheral nerve. Post-intervention results were categorized as improvement, no change, or worsening of symptoms and extracted from both subjective and objective outcome measures. Subjective pain outcomes included the Visual Analogue Scale (VAS: 0–10) and a global perceived change in pain (improved, no change, or worsened. Subjective sensory deficits were assessed using the Medical Research Council Sensory grade (MRCS: S0–S4), the LENT-SOMA scale, and descriptive changes in using static two-point discrimination. Objective outcome measures included muscle strength assessed using the Medical Research Council muscle grade (MRC: M0–M5) and range of motion (ROM).

A formal risk of bias assessment was not performed, as all included studies were descriptive case reports or case series. These study designs are inherently associated with a low level of evidence and substantial risks of selection, reporting, and publication bias.

Due to the clinical and methodological heterogeneity across the included studies and their descriptive study designs, a meta-analysis was not feasible. Therefore, the findings were synthesized narratively, with descriptive statistics reported where applicable. Study characteristics and outcomes were summarized in tables and figures.

## 3. Results

### 3.1. Study Selection

**Figure 1:**
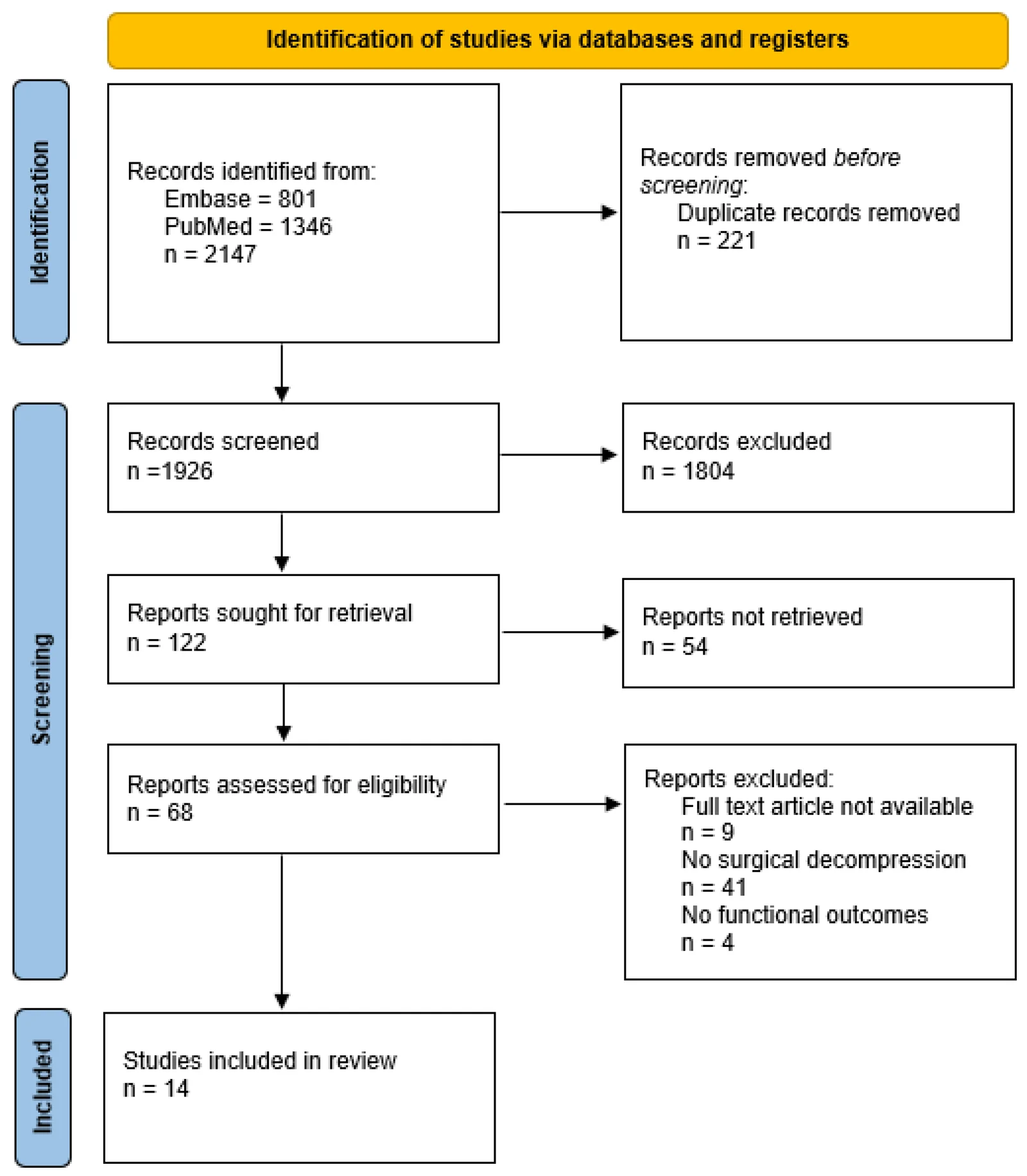
Study selection flowchart.

After removal of duplicates, a total of 1926 citations were identified in PubMed and Embase databases. Following title and abstract screening, potentially relevant articles were selected for full-text review, resulting in 14 studies included in the qualitative synthesis (Figure 1). Two studies evaluated the outcomes of surgical decompression in patients with chemotherapy-induced peripheral neuropathy [16,17], while the remaining twelve focused on radiation-induced peripheral neuropathy [18,19,20,21,22,23,24,25,26,27,28,29].

#### 3.1.1. Study Characteristics

All included studies were case studies or series, comprising a total of 95 patients and 120 intervened nerves (Table 1). Across the included studies, peripheral neuropathy was typically diagnosed following neurological consultation and electrodiagnostic studies. Patients who did not respond to conservative treatment were subsequently treated surgically. Staging of neuropathy severity was performed using a pressure-specified sensory device, an electromechanical computer-assisted measuring device traceable to the National Institute for Standards and Testing. All patients had undergone treatment for malignant tumors, with breast carcinomas most frequently reported. Of all surgical interventions, 23.3% were performed in the upper extremity and 76.7% in the lower extremity. Surgical intervention was generally performed in patients presenting with motor impairment leading to functional limitations in activities of daily living, persisting pain despite conservative pain treatment, sensory deficits, and paresthesia. A wide variation in functional outcome measurements was used, of which VAS, static two-point discrimination, and MRC were most commonly used. In all studies, only one complication in the form of infection was described as a result of surgical intervention.

#### 3.1.2. Characteristics Chemotherapy-Induced Peripheral Compression Neuropathy

Two studies [16,17] evaluated the effectiveness of microsurgical decompression and neurolysis for chemotherapy-induced peripheral compression neuropathy in a total of 11 patients, of whom eight underwent surgical intervention involving 26 nerves (Table 2). Chemotherapy received by patients included cisplatin (*n* = 4), paclitaxel (*n* = 2), a combination of cisplatin and paclitaxel (*n* = 2), thalidomide (*n* = 2), and vincristine (*n* = 1). At presentation, all patients demonstrated a positive Tinel’s sign on clinical examination, indicative of focal nerve compression. Most exhibited grade 3 chemotherapy-induced neuropathy according to the Eastern Cooperative Oncology Group scale, characterized by severe sensory loss, paraesthesia, and disabling pain despite conservative management. The mean age at surgery was 54 years, and six patients (75%) were female. Underlying malignancies included ovarian cancer (*n* = 2), breast cancer (*n* = 2), lung cancer (*n* = 1), lymphoma (*n* = 1), myeloma (*n* = 1), and Behçet’s disease (*n* = 1). The latency between chemotherapy completion and symptom onset ranged up to five years (*n* = 1), while surgery was typically performed more than one year after cessation of treatment. Median follow-up was 9.6 years (range 0.5–12 years).

The majority of these interventions (92.3%) were performed on the lower extremity, primarily targeting the tibial and peroneal nerves, while 7.7% addressed upper-extremity nerves. Decompression alone was performed in 69.2% (*n* = 18) of nerves and combined decompression with neurolysis in 30.8% (*n* = 8).

Postoperative outcomes were consistently favorable, as all patients reported improvement in both pain and sensory deficits. Median Visual Analogue Scale (VAS) scores improved from 9.6 (IQR 8–10) preoperatively to 1.0 (IQR 0–2) postoperatively. Two-point discrimination improved in all cases, with partial or complete recovery of discriminatory sensibility, although no correlation was found between improvements in pain and sensibility. No study assessed motor function or range of motion as formal outcomes, and no postoperative deterioration was reported.

Dellon et al. [16] described six patients who underwent surgical multilevel decompression, including tibial, peroneal, and radial nerves. Three patients who underwent decompression for median, radial, and tibial nerve entrapment were excluded from the analysis due to death secondary to disease recurrence. Chemotherapeutic agents implicated included paclitaxel, cisplatin, vincristine, and combination regimens. All patients experienced reduction in pain and improvement in two-point discrimination, which normalized in two patients. Rose et al. [17] reported two patients treated with bilateral peroneal and tibial nerve decompression combined with neurolysis of unspecified type, for thalidomide-induced neuropathy secondary to myeloma and Behçet’s disease. Surgery consisted of nerve decompression and neurolysis of the common peroneal nerve at the fibular neck, the deep peroneal nerve on the dorsum of the foot, and the tibial nerve in the tarsal tunnel. Both achieved pain relief and improvements in two-point discrimination; one-point discrimination normalized in one patient.

#### 3.1.3. Characteristics Radiation-Induced Peripheral Compression Neuropathy

Twelve studies [18,19,20,21,22,23,24,25,26,27,28,29] evaluated the effectiveness of microsurgical treatment for peripheral compression neuropathies induced by radiotherapy in a total of 93 patients, of whom 87 underwent surgical intervention involving 89 nerves (Table 2). The mean age was 55.6 years, and 83 patients (97%) were female. The underlying malignancies were predominantly breast cancer (*n* = 78, 90.7%), followed by Hodgkin lymphoma (*n* = 3, 3.5%) and single cases of parotid gland, bladder, desmoid, osteosarcoma, and nasopharyngeal cancers. The mean latency between radiotherapy and symptom onset was 6.6 years, and the mean interval between radiation and surgery was 8.2 years. The mean follow-up was 43.6 months.

Nearly all interventions (98.3%) involved the brachial plexus; only two (1.7%) were performed in the lower extremity. Across all studies, 77 patients (88.5%) reported improvement in pain, 40 (45.9%) in sensory function, 43 (49.4%) in MRC, and 18 (20.7%) in range of motion. Postoperative deterioration occurred in 24 patients (27.6%), most commonly affecting motor or sensory function.

#### 3.1.4. Neurolysis with Vascularized Soft Tissue Reconstruction

Two case series [18,22] and one case report [25] evaluated the outcomes of neurolysis combined with either omentoplasty (*n* = 37) or local skin flaps (*n* = 3), comprising a total of 40 patients. This approach aimed to relieve pain and prevent further progression of paresis. Brunelli et al. [18] reported that external neurolysis was sufficient to release the nerves in the majority of cases, whereas internal neurolysis was performed only in patients with more extensive perineural sclerosis. Among the 34 patients who underwent neurolysis combined with vascularized soft tissue reconstruction, consisting of free microvascular transfer of the greater omentum or a local rotational skin flap, the proportions undergoing external versus internal neurolysis were not specified. Killer et al. [22] reported four patients treated with combined internal and external neurolysis followed by omentum grafting. Oliveira et al. [25] described a single case in which extensive external neurolysis was combined with free omentum transfer. Reported outcomes were favorable:97.5% of patients experienced pain relief, sensory deficits improved in 55%, and motor function, assessed using the MRC scale, improved in 45%. Conversely, sensory function deteriorated in 12.5% of patients and MRC grade decreased in 5%.

#### 3.1.5. Neurolysis

The outcomes of neurolysis alone were described in seven case reports and case series comprising 36 patients, with more variable results reported across studies [18,20,21,22,23,24,27]. Brunelli et al. [18] described the outcomes of external and internal neurolysis in three cases; however, the proportions of patients undergoing each technique were not specified. Gosk et al. [20] described external neurolysis of the nerves in the axilla and the superior trunk of the brachial plexus in five patients. Killer et al. [22] reported external neurolysis in four patients. Warade et al. [27] reported that external neurolysis was performed in all 11 patients, which consisted of excision of the perineural fibrosis and scar tissue surrounding the brachial plexus nerves. Kibici et al. [21] treated 11 patients and reported external neurolysis in their methods section, whereas internal neurolysis was reported in their discussion. Mendes et al. [23] reported internal neurolysis of the femoral nerve in one patient. Nich et al. [24] reported neurolysis combined with epineurotomy of the median nerve trunk in the brachial and axillary region in one patient. Pain improvement was reported in 72.5% of cases, with partial sensory recovery in 44% and motor improvement in 21%. Deterioration was observed in sensory function (3%) and MRC grade (27%) (Table 3).

#### 3.1.6. Nerve Reconstruction

Nerve reconstruction procedures including neurolysis with grafting or nerve transfer were described in three case reports (*n* = 10) [26,28,29]. This approach primarily aimed to restore range of motion and muscle strength. Overall, 70% experienced pain reduction, 10% improved in sensory function, and all patients demonstrated gain in muscle strength without deterioration of symptoms. Indications included incomplete denervation of the neuromuscular endplate, preserved donor fascicles, and the absence of tumor infiltration.

Tung et al. [26] achieved recovery of MRC 4/5 elbow flexion and complete pain resolution after median- and ulnar-to musculocutaneous fascicle transfer, twelve years after radiation therapy for breast cancer. Wong et al. [28] reported improved elbow flexion and shoulder abduction with improvements of neuropathic pain following partial ulnar-to-bicep-nerve-transfer, but had persistent C6–C7 dermatomal numbness. Yin et al. [29] demonstrated successful reinnervation in seven of eight patients after sural nerve grafting to the musculocutaneous nerve after segmental resection, with mean VAS decreasing from 2.6 to 0.6.

#### 3.1.7. Free Functioning Muscle Transfer

Free functioning muscle transfer (FFMT) was reported in a single case study [19] involving a patient with radiation-induced brachial plexopathy presenting with profound motor loss but no pain. A Gracilis FFMT, with motor nerve coaptation to the spinal accessory nerve, was performed to restore elbow flexion and improve hand prehension. Isolated nerve transfer was not feasible, as neither the biceps nor brachialis contracted on stimulation, reflecting longstanding palsy and axonal injury. At two-year follow-up, elbow flexion range of motion had improved from 40 to 110, accompanied by enhanced hand function and reduction in the Disabilities of the Arm, Shoulder and Hand (DASH) score from 56 to 20.

## 4. Discussion

This systematic review evaluated the outcomes of microsurgical decompression for peripheral compression neuropathies secondary to chemotherapy (CIPN) (2) and radiotherapy (RIPN (12)). Across all studies, patients experienced persistent, refractory pain or sensory deficits despite comprehensive conservative management. Overall, our findings indicate that decompression surgery may provide meaningful symptom relief and partial functional recovery in selected patients with treatment-related superimposed neuropathies. Pain, measured by the VAS score, and sensory function, assessed by two-point discrimination, improved consistently following surgical intervention. These outcomes support the concept that, in both CIPN and RIPN, secondary superimposed compression contributes to the severity of neuropathic symptoms, and that surgical release of these entrapments might reduce pain and restore sensory transmission.

### 4.1. Chemotherapy-Induced Peripheral Compression Neuropathy

The proposed pathophysiological mechanisms underlying CIPN suggest that chemotherapy-induced neurotoxicity increases the susceptibility of peripheral nerves to secondary compression at anatomical sites of entrapment. Direct neurotoxic effects are most prominently associated with platinum compounds, taxanes, vinca alkaloids, and proteasome inhibitors, resulting in axonal injury, loss of intraepidermal nerve fibers, and neuroinflammatory changes associated with increased neuronal excitability, resembling mechanisms observed in traumatic and diabetic neuropathies. Indirect toxicity may result from treatment-related tissue changes, including edema, neuroinflammation, and potentially fibrotic remodeling of surrounding tissues, which may further contribute to peripheral nerve compression and secondary entrapment syndromes [30,31]. Although the precise mechanisms remain only partially understood and vary between agents, chemotherapy impairs both axonal transport and microvascular integrity, increasing their vulnerability to secondary compression at anatomically constrained sites [32]. These mechanisms may be particularly relevant in the lower extremities, as CIPN is typically length-dependent and predominantly affects distal peripheral nerves. When a pre-existing distal neuropathic insult is combined with a second focal compressive lesion, symptoms may become disproportionate in severity and persistence [33,34]. The clinical manifestation and severity of CIPN depend on the specific agent, cumulative dose, treatment duration, and concomitant exposure to other neurotoxic drugs [35].

Surgical intervention may be most appropriate in patients with demonstrable focal nerve compression, characterized by a positive Tinel’s sign, localized pain patterns at known entrapment sites, and supportive diagnostic findings [13,32,33,34,35,36,37,38,39,40,41]. Predictors of surgical success remain incompletely defined, but several factors appear to be relevant. A positive Tinel’s sign, indicative of ongoing axonal degeneration and regeneration, has been associated with both greater baseline neuropathic pain and improved postoperative pain relief following decompression [37,42,43]. Conversely, in advanced disease, progressive demyelination and loss of regenerative capacity may result in an absent Tinel’s sign, thereby reducing its prognostic value [44]. Similarly, preserved two-point discrimination, reflecting preserved axonal density and residual functional sensory fibers, correlates with better postoperative outcomes, whereas absent discrimination suggests advanced axonal loss and poorer recovery potential.

The diagnostic reliability of electrophysiological testing in this context remains limited. Diffuse axonal injury from chemotherapy predominantly reduces sensory and motor response amplitudes, with variable effects on conduction velocity, thereby limiting the sensitivity and specificity of nerve conduction studies [45,46,47]. As a result, diagnosis and assessment of surgical candidacy do not rely solely on electrodiagnostic studies, but should integrate clinical findings, symptom distribution, and patient history. Nerve ultrasound studies suggest that CIPN may be accompanied by altered nerve morphology, including increased cross-sectional area (CSA) in selected peripheral nerves, particularly at common entrapment sites [48,49,50,51]. However, these findings are heterogeneous across agents and studies. Oxaliplatin-induced neuropathy has been associated with nerve enlargement at entrapment sites in both upper and lower extremity nerves. Taxane-induced neuropathy demonstrated more variable findings, including reduced sural nerve size and mild CSA enlargement of the median nerve at the distal wrist crease. These observations support the possibility that chemotherapy-related nerve changes may increase susceptibility to secondary compression.

In the two available studies, patients with CIPN undergoing decompression reported reductions in pain (mean VAS decrease of 7.6) and improvement in two-point discrimination. All exhibited a positive Tinel’s sign at the affected sites, consistent with clinical signs of focal nerve entrapment. While CIPN is currently managed almost exclusively with conservative strategies [52], these findings suggest that surgical decompression may represent a rational therapeutic option for carefully selected patients with evidence of superimposed focal nerve entrapment, a positive Tinel’s sign and preserved two-point discrimination. Experimental data from cisplatin-induced neuropathy models have demonstrated similar benefits, reinforcing the possible effectiveness of this intervention [53].

An additional observation was the absence of specifically reported outcomes for median or ulnar nerve decompression. Notably, two patients who underwent decompression of the median nerve in one of the included studies were excluded from the analysis because they died from recurrent disease. In contrast, no cases of ulnar nerve decompression were reported in the included studies.

### 4.2. Radiation-Induced Peripheral Compression Neuropathy

RIPN represents a delayed complication of radiotherapy that may contribute to persistent pain and functional impairment in cancer survivors. First described by Stoll and Andrews in 1966 [54], RIPN arises through both direct neural injury and secondary tissue changes. Direct mechanisms include axonal damage, demyelination, and radiation-induced vascular injury, resulting in ischemia, impaired nerve function, and subsequent axonal degeneration. Indirect mechanisms involve radiation-induced fibrosis and remodeling of surrounding tissues, which may contribute to mechanical compression of peripheral nerves and the development of secondary entrapment syndromes [5,55]. The risk and severity of RIPN correlate with total radiation dose, fraction size, irradiated volume, and treatment technique, while the latency to symptom onset typically ranges from one to four years after therapy, but may extend beyond that interval [10].

Among radiation-induced peripheral neuropathies, radiation-induced brachial plexopathy is the most clinically significant manifestation, typically occurring after irradiation of the breast, axilla, or thoracic outlet [10,56]. This predilection may, in part, relate to the anatomical course of the brachial plexus through relatively confined regions with limited soft-tissue compliance, where radiation-induced fibrosis and tissue swelling may more readily result in mechanical constriction. Earlier series reported incidences exceeding 20%, largely attributable to high-dose, large-field irradiation using conventional techniques [52]. The transition to conformal and intensity-modulated radiotherapy has markedly reduced the risk, with contemporary rates below 2% [53,57].

The development and severity of radiation-induced peripheral neuropathy depend on both treatment-related factors, including cumulative dose, fraction size, field volume, and dose heterogeneity, and patient-related comorbidities, such as diabetes, hypertension, and prior chemotherapy exposure [49,56]. Symptom onset is often delayed, emerging several years after radiotherapy, complicating recognition and attribution. Diagnosis relies on detailed neurological examination, including contralateral comparison, supported by adjunct investigations such as electromyography and magnetic resonance neurography, which help localize the lesions and exclude tumor recurrence [11,53,58,59,60].

Once established, RIBP is typically progressive and refractory to conservative therapy, leading to chronic pain, weakness, and, in advanced stages, complete loss of limb function. The prognosis remains poor, and current management is largely symptomatic [27]. The threshold for surgical intervention remains undefined, though some authors have proposed reserving surgery for LENT-SOMA [61] grade 3–4 neuropathies with progressive motor loss or refractory pain [62].

In the present review, surgical decompression was associated with pain improvement in up to 88% of patients, sensory improvement in approximately 59%, and motor recovery in 44%. Among specific techniques, neurolysis combined with vascularized flap reconstruction yielded the most favorable outcomes, with pain relief in nearly all cases and partial restoration of sensory and motor function. In contrast, neurolysis alone achieved more modest results and carried a higher risk of postoperative deterioration in strength, suggesting that adequate soft tissue vascularized coverage may be important to prevent recurrent fibrosis and compression. Nerve transfers and free functional muscle transfers represent potential reconstructive strategies for patients presenting with deficits in range of motion and muscle strength, achieving consistent improvements in pain and motor function. Consistent improvements in pain and motor function were reported when viable neuromuscular endplates were preserved, as confirmed by electromyography. In patients without preserved endplate viability, FFMT and Steindler flexorplasty were alternative procedures described to restore elbow flexion and improve limb positioning, although neurological recovery remained limited. These findings underscore the importance of electrophysiological evaluation before reconstructive planning to identify candidates likely to benefit from nerve-based interventions.

The anatomical distribution of RIPN in the included studies was predominantly characterized by brachial plexopathy, with the majority of cases occurring after radiotherapy for breast cancer. Nevertheless, individual peripheral nerve involvement was also reported, including the femoral, median, ulnar, and musculocutaneous nerves, indicating that radiation-induced peripheral compression neuropathy is not restricted to the brachial plexus.

### 4.3. Strengths and Limitations

Several limitations of this study should be acknowledged. Although the findings suggest a potential role for surgical intervention in selected patients, the available evidence is constrained by methodological limitations of the included studies, and the results should therefore be interpreted with appropriate caution. Evidence regarding the surgical treatment of chemotherapy-induced peripheral compression neuropathy and radiation-induced peripheral compression neuropathy remains limited and is derived exclusively from retrospective case reports, case series, and small cohort studies, with a substantial risk of selection and reporting bias. Considerable heterogeneity was observed across studies with respect to patient characteristics, underlying malignancies, oncological treatment regimens, timing of surgical intervention, surgical techniques, and outcome assessment, precluding quantitative synthesis and limiting cross-study comparability. Outcome reporting was frequently non-standardized and primarily focused on pain relief and sensory improvement, whereas postoperative motor recovery and ROM were rarely evaluated systematically. In addition, the predominance of female patients across both populations restricts the generalizability of the findings.

A further limitation of this review concerns the heterogeneous and imprecise use of the term “neurolysis” across the included studies. Neurolysis may refer to external neurolysis, involving the release of scar tissue and adhesions in the plane between the paraneurium and external epineurium while leaving the epineurium intact, or to internal neurolysis, in which the epineurium is opened to release intraneural adhesions or constrictions. These techniques differ substantially in invasiveness, potential for iatrogenic fascicular injury, and indications. The type of neurolysis performed was explicitly reported in seven of nine included studies reporting neurolysis. External neurolysis alone was reported in three studies, both external and internal neurolysis were differentiated within the same cohort in two studies, and internal neurolysis was described in two further studies. In one of the studies reporting both techniques, the outcomes according to neurolysis technique could not be distinguished, precluding attribution of outcomes to either technique. Notably, one study used the terms external and internal neurolysis inconsistently within the same publication. One study did not provide further specification beyond the term “neurolysis”. Furthermore, operational definitions were rarely provided, making it uncertain whether external or internal neurolysis consistently corresponded to the same extent of dissection across studies. This lack of standardized terminology and reporting limits the comparability of surgical techniques and outcomes between studies and may contribute to the variability in reported outcomes.

The anatomical distribution of nerve involvement should also be interpreted with caution. The small number of published cases, limited number of eligible patients, selective nature of the surgical literature, and study-specific exclusions may have resulted in underrepresentation of individual nerve involvement. Consequently, the predominance of radial, tibial, and peroneal nerve involvement in CIPN and brachial plexopathy in RIPN observed in this review should not be interpreted as reflecting the relative incidence or susceptibility of individual nerves.

Several limitations were specific to the individual conditions. In the CIPN studies, patients often underwent decompression at multiple anatomical sites or in multiple limbs, making it difficult to attribute outcomes to individual procedures. Surgical management predominantly involved decompression of lower-extremity nerves, particularly the tibial and peroneal nerves. In contrast, the RIPN literature was dominated by patients with radiation-induced brachial plexopathy following treatment for breast carcinoma. Furthermore, the often prolonged latency between radiotherapy and symptom onset introduces diagnostic uncertainty, including the possibility of alternative causes of neuropathic pain.

Nevertheless, several strengths should be recognized. To our knowledge, this study represents one of the first structured syntheses of the evidence regarding microsurgical treatment for cancer therapy-induced peripheral compression neuropathies. The review was conducted using a systematic and reproducible methodology, including a comprehensive search strategy across two major databases, predefined eligibility criteria, dual-stage screening, and structured extraction of key demographic, clinical, and surgical characteristics. These methods allowed a structured evaluation of a heterogeneous and currently poorly characterized field. In addition, by systematically mapping the current evidence base, this review identifies critical knowledge gaps and delineates priorities for future prospective studies.

Overall, both CIPN and RIPN remain poorly characterized entities. Future research should prioritize prospective multicenter studies using uniform diagnostic criteria, standardized outcome measures, and longer follow-up to better define the incidence, appropriate surgical indications, and to clarify the role of microsurgical decompression. Improved patient selection, supported by clinical assessment and advanced imaging modalities such as MR neurography and high-resolution nerve ultrasound, may facilitate the development of objective treatment endpoints and more reliable evaluation of surgical outcomes.

## 5. Conclusions

Treatment-related peripheral compression neuropathies may represent an underrecognized contributor to persistent pain and disability following chemotherapy and radiotherapy. In selected patients with progressive, treatment-refractory symptoms and evidence of superimposed focal nerve entrapment, characterized by a positive Tinel’s sign or localized pain patterns at known sites of nerve compression and supported by additional diagnostic investigations, microsurgical decompression may provide meaningful symptomatic and functional benefit. Nevertheless, current evidence remains limited.

## Figures and Tables

**Table 1:** Characteristics of included studies.

Study, Year	*N*	Sex		Age (Y)	Nerve		Tumor Location	Tumor Treatment		Total Dose (Average)/Agent		Onset of Symptoms (Y)	TTI (Y)	Final Outcome Measure	Comp
		F	M		UE	LE		Rx	Cx	Rx	Cx				
Brunelli et al. 1985 [18]	37	37	0	-	37	0	Breast	36	0	NA	-	NA	NA	VAS, Palsy, Radiodermatitis, Sensation, ROM	Nm
Dellon et al. 2004 [16]	9	8	1	59	5	18	Breast Ovarian Lung lymphoma	0	9	-	Cisplatin Paclitaxel Vincristine	NA	1	VAS, 2PD	0
Gangurde et al. 2014 [19]	1	1	0	56	1	0	Breast	1	0	400 Gy	-	7	16	ROM	Nm
Gosk et al. 2007 [20]	5	5	0	57	5	0	Breast Parotid gland	5	3	50 Gy	NA	7.5	10	VAS, sensory deficits, motor deficits	Nm
Kıbıcı et al. 2020 [21]	11	11	0	43	11	0	Breast	11	9	NA	NA	1	1	VAS, 2PD, motor function	Nm
Killer et al. 1990 [22]	9	7	2	69	9	0	Breast Hodgkin lymphoma	12	0	NA	-	4.2	4	VAS, paresis	Nm
Mendes et al. 1991 [23]	1	0	1	55	0	2	Urinary bladder	1	0	27.5 Gy	-	0.3	0.3	VAS, paresthesias, sensory and motor function	Nm
Nich et al. 2005 [24]	1	1	0	38	1	0	Osteosarcoma	1	1	70 Gy	Methotrexate Cyclofosfamide Adriamycin	22	22	VAS, paresthesia, sensitivity	Nm
De Oliveira et al. 2020 [25]	1	1	0	68	1	0	Breast	1	1	NA	NA	18	19	VAS, motor strenght	Nm
Rose et al. 2006 [17]	2	0	2	64	0	8	Behçet Myeloma	0	2	-	Thaliomide	2.5	2.5	VAS, 2PD	Inf (1)
Tung et al. 2009 [26]	1	1	0	59	2	0	Breast	1	0	48 Gy	-	3	3	MRC, FDA function	Nm
Warade et al. 2019 [27]	11	11	0	48	11	0	Breast	11	11	53 Gy	NA	1.6	2.3	VAS, paresthesia	Nm
Wong et al. 2009 [28]	1	1	0	65	1	0	Breast	1	1	60 Gy	NA	0.7	1.8	MRC, pain, 2PD	Nm
Yin et al. 2022 [29]	8	8	0	53	8	0	Breast Nasopharyngeal Desmoid	8	0	53 Gy	-	8	9.8	VAS, MRC	0

2PD; Two-point discrimination, Cx; Chemotherapy, Comp; Complicatons, F; Female, Inf; Infection, LE; Lower extremity, M; Male, N; Number of patients, NA; Not available, Nm; Not mentioned, ROM; Range of motion, Rx; Radiotherapy, TTI; Time between therapy and intervention in years, UE; Upper extremity, VAS; Visual analogue scale.

**Table 2:** Characteristics and outcomes of surgical decompression in CIPN.

Study, Year	*N*	Intervention		Location			Post-Intervention Symptom Improvement				Post-Intervention Symptom Worsening				FU (Months)
		Decompression	Decompression with Neurolysis				Subjective		Objective		Subjective		Objective		
				N. radialis	N. peroneus	N. tibialis	VAS	Sensory deficit	MRC	ROM	VAS	Sensory deficit	MRC	ROM	
Dellon et al. 2004 [16]	6	18	0	2	8	8	18	18	NA	NA	NA	NA	NA	NA	129.6
Rose et al. 2006 [17]	2	0	8 *	0	4	4	8	8	NA	NA	NA	NA	NA	NA	9

*; Neurolysis not specified. FU; Follow-up, N; Number of patients, NA; Not available, ROM; Range of motion, VAS; Visual analogue scale.

**Table 3:** Characteristics and outcomes of surgical decompression in RIPN.

Study	Intervention				Nerve Treated	Post-Intervention Symptom Improvement				Post-Intervention Symptom Worsening				Follow-Up (Month)
						Subjective		Objective		Subjective		Objective		
	Nwvf	Neu	NG	FFMT		VAS	Sensory deficit	MRC	ROM	VAS	Sensory deficit	MRC	ROM	
Brunelli et al. 1985 [18]	3 † *				BP	2	0	NA	1	0	3	NA	3	51
	31 *				BP	31	22	18	2	0	2	1	3	51
Killer et al. 1990 [22]	5 *				BP	5	NA	NA	NA	0	NA	NA	NA	168
De Oliveira et al. 2020 [25]	1 **				BP	1	NA	0	NA	0	NA	1	NA	6
Brunelli et al. 1985 [18]		3 *			BP	2	0	NA	0	1	1	NA	3	51
Gosk et al. 2007 [20]		5 **			BP	3	3	2	NA	0	0	0	NA	NA
Kıbıcı et al. 2020 [21]		11 ****			BP	11	3	3	NA	0	0	0	NA	6
Killer et al. 1990 [22]		4 **			BP	2	NA	NA	NA	0	NA	NA	NA	171
Mendes et al. 1991 [23]		1 ***			Femoral	1	1	1	NA	0	0	0	NA	5
Nich et al. 2005 [24]		1 ***			BP	1	1	NA	NA	0	0	NA	NA	12
Warade et al. 2019 [27]		11 **			BP	11	9	2	NA	0	0	8	NA	11
Tung et al. 2009 [26]			1		Ulnar and median	1	0	1	NA	0	0	0	NA	24
Wong et al. 2009 [28]			1		Ulnar	1	1	1	NA	0	0	0	NA	24
Yin et al. 2022 [29]			8		Musculocutaneous	5	NA	8	8	0	NA	0	0	32
Gangurde et al. 2014 [19]				1	BP	NA	NA	1	NA	NA	NA	0	NA	24

†; Vascularized skin flap, *; External and internal neurolysis, **; External neurolysis, ***; Internal neurolysis, ****; Neurolysis inconsistently reported, BP; Brachial plexus, FFMT; Free functioning muscle transplantation, NA; Not available, NG; Nerve grafting, Neu; Neurolysis, NwvF; Neurolysis with vascularized flap.

## Data Availability

No new data were created or analyzed in this study. Data sharing is not applicable to this article.

## Supplementary Materials

The following supporting information can be downloaded at https://www.mdpi.com/article/10.3390/jcm15176538/s1. Supplementary File S1: PRISMA abstract checklist; Supplementary File S2: PRISMA checklist; Supplementary File S3: Complete search syntax.

## Abbreviations

The following abbreviations are used in this manuscript:

CIPN	Chemotherapy-induced peripheral neuropathy
CSA	Cross-sectional area
FFMT	Free functioning muscle transfer
IQR	Interquartal range
MRC	Medical Research Council muscle grade
MRCS	Medical Research Council sensory grade
RIBP	Radiation-induced brachial plexopathy
RIPN	Radiation-induced peripheral neuropathy
ROM	Range of motion
TLA	Three-letter acronym
VAS	Visual Analogue Scale
