# Supplementary material for: Decompressive Surgery in Chemotherapy- and Radiotherapy- Induced Peripheral Compression Neuropathy: A Systematic Review
Source: J Clin Med. 2026 Aug 24;15(17):6538. doi: 10.3390/jcm15176538 (PMC13566654; doi:10.3390/jcm15176538)
Supplement: Supplementary file 1 [file jcm-15-06538-s001.zip › Supplementary File S3. Search syntax.pdf]

S.3.1 PubMed

("chemotherapy, adjuvant"[MeSH Terms] OR "Neoplasms/drug therapy"[MeSH Terms] OR "Antineoplastic agents/adverse effects"[MeSH Terms] OR "Radiotherapy"[MeSH Terms] OR "Neoplasms/Radiotherapy"[MeSH Terms] OR "Chemo"[Title/Abstract] OR "Antineoplastic therap*"[Title/Abstract] OR "Cytotoxic therap*"[Title/Abstract] OR "Onco* therap*"[Title/Abstract] OR "System* therap*"[Title/Abstract] OR "Radio*"[Title/Abstract] OR "Therapeutic radiolog*"[Title/Abstract] OR "Irradiation therap*"[Title/Abstract] OR "External beam radiation therap*"[Title/Abstract] OR "brachytherapy*"[Title/Abstract] OR "Combined modality therap*"[Title/Abstract]) AND ("Small fiber neuropathy"[MeSH Terms] OR "Peripheral nervous system diseases"[MeSH Terms] OR "Peripheral nerve injuries"[MeSH Terms] OR "Radiation injuries*/surgery"[MeSH Terms] OR "Peripheral nerve injur*"[Title/Abstract] OR "Neuropath*"[Title/Abstract] OR "Nerve damage*"[Title/Abstract] OR "Neurodegeneration*"[Title/Abstract] OR "Nerve disorder*"[Title/Abstract]) AND ("Decompression, Surgical"[MeSH Terms] OR "Nerve Compression Syndromes/Surgery"[MeSH Terms] OR "Nerve decompression*"[Title/Abstract] OR "Decompression surg*"[Title/Abstract] OR "Peripheral nerve release*"[Title/Abstract] OR "Nerve release surg*"[Title/Abstract] OR "Decompressive surg*"[Title/Abstract] OR "Neurolysis"[Title/Abstract] OR "Neural decompression*"[Title/Abstract])*

S.3.2 Embase

('adjuvant chemotherapy'/exp OR 'neoplasm'/exp/dm_dt OR 'antineoplastic agent'/exp/dd_ae OR 'radiotherapy'/exp OR 'neoplasm'/exp/dm_rt OR 'chemo':ti,ab,kw OR 'antineoplastic therap*':ti,ab,kw OR 'cytotoxic therap*':ti,ab,kw OR 'on-co* therap*':ti,ab,kw OR 'system* therap*':ti,ab,kw OR 'ra-dio*':ti,ab,kw OR 'therapeutic radiolog*':ti,ab,kw OR 'irradiation therap*':ti,ab,kw OR 'external beam radiation therap*':ti,ab,kw OR 'brachytherapy*':ti,ab,kw OR 'combined modality therap*':ti,ab,kw) AND ('small fiber neuropathy'/exp OR 'peripheral neuropathy'/exp OR 'peripheral nerve injury'/exp OR 'radiation inju-ries*'/exp/dm_su OR 'peripheral nerve injur*':ti,ab,kw OR 'neuro-path*':ti,ab,kw OR 'nerve damage*':ti,ab,kw OR 'neurodegenera-tion*':ti,ab,kw OR 'nerve disorder*':ti,ab,kw) AND ('decompression, sur-gical'/exp OR 'nerve compression'/exp/dm_su OR 'nerve decompression*':ti,ab,kw OR 'decompression surg*':ti,ab,kw OR 'peripheral nerve release*':ti,ab,kw OR 'nerve release surg*':ti,ab,kw OR 'decompressive surg*':ti,ab,kw OR 'neurolysis':ti,ab,kw OR 'neural de-compression*':ti,ab,kw)*
